# Vaccination and mortality from COVID-19: a comparative study between hemodialysis, peritoneal dialysis, and non-dialysis adult population in Panama

**DOI:** 10.1007/s11255-023-03529-w

**Published:** 2023-02-24

**Authors:** Karen J. Courville, Norman E. Bustamante, Virginia Nuñez-Samudio, Maydelin Pecchio, Iván Landires, Carlos Viggiano, Herna Durán, Nadji Novoa, Ernesto Alvarado, Francisco Vargas, Dayan Salado, José Manzanares, Kelly Haughton, César Cuero, María Niedda, Régulo Valdés

**Affiliations:** 1 Sociedad Panameña de Nefrología e Hipertensión, Calle Gervasio García, Edificio Cetrersa, Piso 3, Hato Pintado, 0819 Panamá, Panamá; 2 Nefrología/Hospital Dr. Gustavo N. Collado, Caja de Seguro Social, Chitré, Panamá; 3 Departamento de Investigación/Instituto de Ciencias Médicas, Las Tablas, Panamá; 4 Nefrología/Complejo Hospitalario Metropolitano, Caja de Seguro Social, Panama City, Panamá; 5 Coordinación Nacional de Hemodiálisis, Panama City, Panamá; 6 Nefrología/Hospital Dr. Rafael Hernández, Caja de Seguro Social, David, Panamá; 7 Nefrología/Policlínica Dr. Horacio Díaz Gómez, Santiago de Veraguas, Panamá; 8 Nefrología/Hospital Ezequiel Abadía, Soná, Panamá; 9 Nefrología/Hospital Dr. Rafael Estevez, Caja de Seguro Social, Aguadulce, Panamá; 10 Nefrología/Policlínica Dr. Santiago Barraza, La Chorrera, Panamá; 11 Nefrología/Unidad de Hemodiálisis Metro 1, Panama City, Panamá; 12 Nefrología/Unidad de Hemodiálisis Metro 2, Panama City, Panamá; 13 Nefrología/Hospital Dra. Susana Jones Cano, San Miguelito, Panamá

**Keywords:** COVID-19, Dialysis, Hemodialysis, Peritoneal dialysis, Vaccines

## Abstract

**Purpose:**

Dialysis patients have a different response than the non-dialysis population to infection with COVID-19. This study evaluates the prevalence of infection and lethality in patients receiving hemodialysis or peritoneal dialysis in Panama, compared to non-dialysis adult population, and reports of adverse events of vaccination.

**Methods:**

This is a prospective, multi-center cohort study of spatients aged 18 years or older and receiving in-center hemodialysis or ambulatory peritoneal dialysis in 13 centers in Panama from March 2021 to 2022. For comparison with general population, the study used an extended period of two years.

**Results:**

A total of 1531 patients receiving dialysis treatment accepted to participate. PD patients represented an 18% of study patients. Lethality was higher in peritoneal dialysis patients with COVID-19 infection than in hemodialysis in the study group (*p* 0.02). Total deaths in dialysis patients for 2020 were 156 patients, before vaccination; 79 in 2021; and 25 for the first trimester of 2022. Lethality for the period of 2020–2022 was 9.3% for dialysis patients and 0.2% for non-dialysis population. There was no difference in symptoms in first dose, but with second dose, hemodialysis patients reported fewer symptoms than peritoneal dialysis patients (*p* < 0.0001).

**Conclusion:**

Ninety one percent of people in the country received BNT162b2 Pfizer BionTech vaccine. Lethality decreased from 30 to 5% once vaccination was available. There were no severe adverse effects and symptoms reported were less frequent than in general population, probably due to low reactogenicity in dialysis patients, or better tolerance to pain.

## Introduction

Since the beginning of the SARS-CoV-2 pandemic, dialysis patients have been recognized as a highly affected group of patients, with mortality levels higher than general population [1]. Availability of COVID-19 vaccines was associated with a reduction in the risk of severe infection, including COVID-19-related hospitalization and death.

Retention of uremic products in dialysis patients produces a continual state of oxidative stress, inflammation, and immunological dysfunction [2]. Studies report that optimal responses to vaccination in this population may depend on their immune senescence or their ability to generate T cells against COVID-19 [3]. Coronavirus disease has presented various mutations during this pandemic and response to different variants can vary among immunocompromised patients, because it is recognized that CKD patients can present a reduction to vaccine response, with a diminished or delayed capacity to generate antibodies [4]. Adverse events in dialysis population after vaccination could be more or less severe, depending on reactogenicity, also related to uremia-associated immunodeficiency and or weakening defense mechanisms [5].

This study of adults receiving three doses of COVID-19 vaccines evaluates whether there is a higher prevalence of infection in patients receiving hemodialysis or peritoneal dialysis in Panama, compared to non-dialysis adult population in the country. The study also compares mortality in dialysis population before vaccine availability and after three dose applications. Finally, the study reports the rate of recognized adverse effects after every vaccination dose.

## Materials and methods

The study reports a prospective, multi-center cohort study of patients aged 18 years or older and receiving in-center hemodialysis or ambulatory peritoneal dialysis in Panama. The goal was to evaluate the clinical response to BNT162b2 COVID-19 vaccine (Pfizer-BioNTech), which was the first vaccine available in Panama in January 2021. The study was done at 13 dialysis centers nationwide. The vaccination schedule approved in February 2021 for chronic kidney disease in hemodialysis or peritoneal dialysis was: a first dose during the month of March 2021 with the second dose 21 days later. A third dose was approved on September 2021, with patients receiving a third BNT162b2 COVID-19 vaccine dose the first week of October. Follow up was done until March 2022. All participants received three doses.

For people without chronic kidney disease on renal replacement therapy (hemodialysis or peritoneal dialysis), the study included all adults aged 19–99 years who had two or three doses of the ChAdOx1 nCoV-19 (Oxford-AstraZeneca) or BNT162b2 (Pfizer-BioNTech) vaccine during the study period until March 31st 2022.

Panama has an ethnic distribution composed of 60% Mestizo (Spanish-Native), 16% Caucasian, 14% Mulatto (afro descendants), 6% Native and 4% Asian population [6]. Patients in hemodialysis centers, and peritoneal dialysis patients in ambulatory visits, were offered to participate in the study. Those who agreed completed electronic questionnaires independently or with the assistance of nursing staff.

All consenting patients receiving hemodialysis in participating centers were included. Hemodialysis patients were treated with in a three-times-a-week schedule of 4 h per session. The study collected information on age, sex, location of hemodialysis center, time in hemodialysis treatment, comorbidities.

Patients on peritoneal dialysis received daily continuous ambulatory or automated peritoneal dialysis. The study collected information about age, sex, location of peritoneal dialysis clinic, time on peritoneal dialysis treatment and comorbidities.

Infections with SARS-CoV-2 were confirmed through real-time RT-PCR assay of nasopharyngeal swab specimens following detection of symptoms (respiratory, sore throat, rhinorrhea, fever) in each dialysis center. The National Dialysis Coordination provided data on total number of patients in dialysis in the Social Security System, reported COVID-19 positive infected patients and deceased patients from March 2020. Social Security System covers 90% of dialysis population in Panama; the remaining 10% is distributed between National Health Ministry units and private dialysis centers.

For a control group that represents the general Panama population, the study used data from the National Authority for Government Innovation (NAGI) COVID-19 vaccination public database [7], which contains information about the type of vaccine, how many doses were administered and demographics of the population, total positive cases and mortality in the general population since March 2020 until March 2022.

For both hemodialysis and peritoneal dialysis patients, information was collected on occurrence and duration of local (redness, pain, and swelling) and systemic side effects (fever, fatigue, headache, muscle pain, joint pain, chills, vomits, and diarrhea) of three dose vaccinations.

The data collected from electronic questionnaires for dialysis patients included the period from March 2021 to March 2022. For evaluation of the effect of constituted immunity after vaccination that takes in average 2–3 weeks, the first period of observation was determined from April 2021 (1 month after first dose in March); second period was determined from October 2021, after the third dose was applied; and the third period for observation, until March 2022. For comparison with general population, the study used an extended period from March 2020 until March 2022.

Data were presented as a percent for categorical variables and median for continuous variables. Data analyses were performed in Stata v. 11.0 (StataCorp, LLC; College Station, TX, USA). Descriptive statistics were calculated, Fisher’s exact test was used to compare proportions, setting the alpha value at 0.05 for statistical significance. Chi-square test was used for categorical variables. Univariable analysis was done to identify factors associated with dialysis risk. The case fatality rate for COVID-19 was calculated using the following equation: number of deaths/total confirmed cases in percentage.

The study was approved by the National Bioethics Committee EC-CNBI-2021-02-134. Informed consent was required for dialysis patients to participate. The investigators designed the trial, wrote the manuscript, and vouch for the accuracy and complete reports of data and/or adverse events in adherence with protocol design. The funding organization was not involved in design, implementation, and analysis of data or decision to submit manuscript for publication.

## Results

**Fig. 1:**
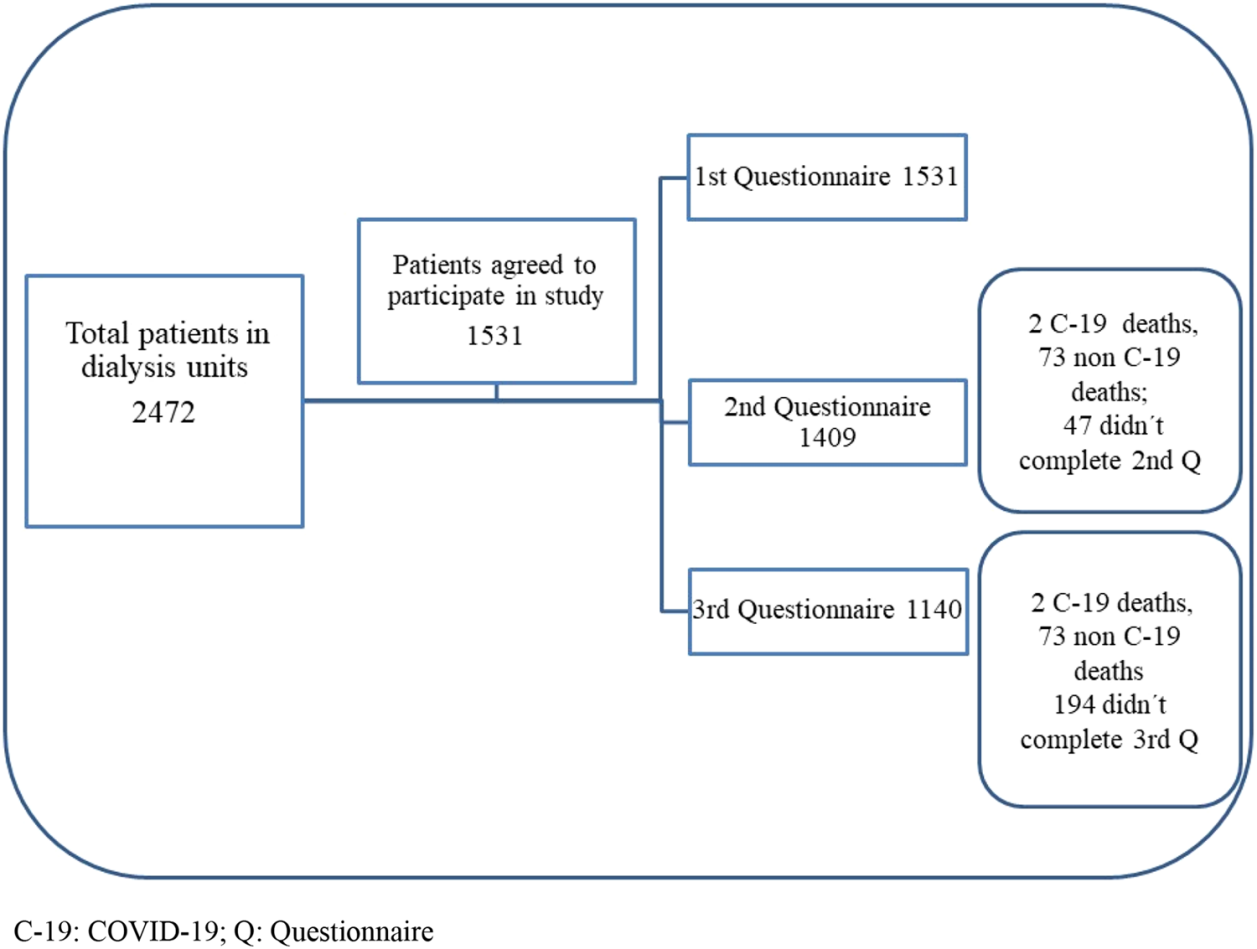
Patients included in the study, deaths, and follow-up

The National Dialysis Coordination reported 2472 patients receiving dialysis on March 2021 (2033 in hemodialysis and 439 in peritoneal dialysis) [8]. A total of 1531 patients receiving dialysis treatment, distributed in 13 units of the Social Security System in the national territory, accepted to participate in the study and completed first electronic questionnaire; 1409 completed the second electronic questionnaire, and 1140 patients completed the third questionnaire. The difference in patients between each survey corresponds to deaths from COVID-19, other infectious, and/or cardiovascular causes, as described in Fig. 1.

Table 1 shows the main characteristics of the participating patients. The majority of the patients were male (61% in hemodialysis and 64% in peritoneal dialysis); most of the patients were grouped in the age groups of 56–70 years in both treatment modalities; there was a difference between the time on dialysis, with 59% of patients on hemodialysis and 26% of patients on peritoneal dialysis, in the group of more than 36 months (*p* = 0.001); and the most frequent comorbidity was hypertension in both groups, followed by diabetes, but with a significant difference in peritoneal dialysis patients (*p* = 0.005).

**Table 1:** General characteristics of study participants

Total patients	*N* = 1531		
Characteristics	Hemodialysis *n* = 1246	Peritoneal dialysis *n* = 285	*p*
Sex	*n* (%)	*n* (%)	0.379
Male	763 (61)	183 (64)	
Female	483 (39)	102 (36)	
Age group (years)			
18–25	30 (2.4)	9 (3)	0.484
26–40	160 (13)	38 (13)	
41–55	363 (29)	76 (27)	
56–70	489 (39.2)	124 (44)	
≥ 71	204 (16.4)	38 (13)	
Time on dialysis (months)			
0 to 3	25 (2)	47 (16.5)	0.001
4 to 12	173 (14)	81 (28.4)	
13 to 36	312 (25)	82 (28.8)	
> 36	736 (59)	75 (26.3)	
Comorbidities			
Hypertension	528 (42)	136 (47.7)	0.112
Diabetes	390 (31)	114 (40)	0.005
Glomerulopathies	195 (16)	10 (3.5)	0.001
Polycystic kidney disease	35 (3)	0 (0)	0.001
Lupus nephritis	22 (1.8)	0 (0)	0.022
Other	33 (2.6)	14 (4.9)	
Unknown	43 (3.5)	11 (3.9)	

**Fig. 2:**
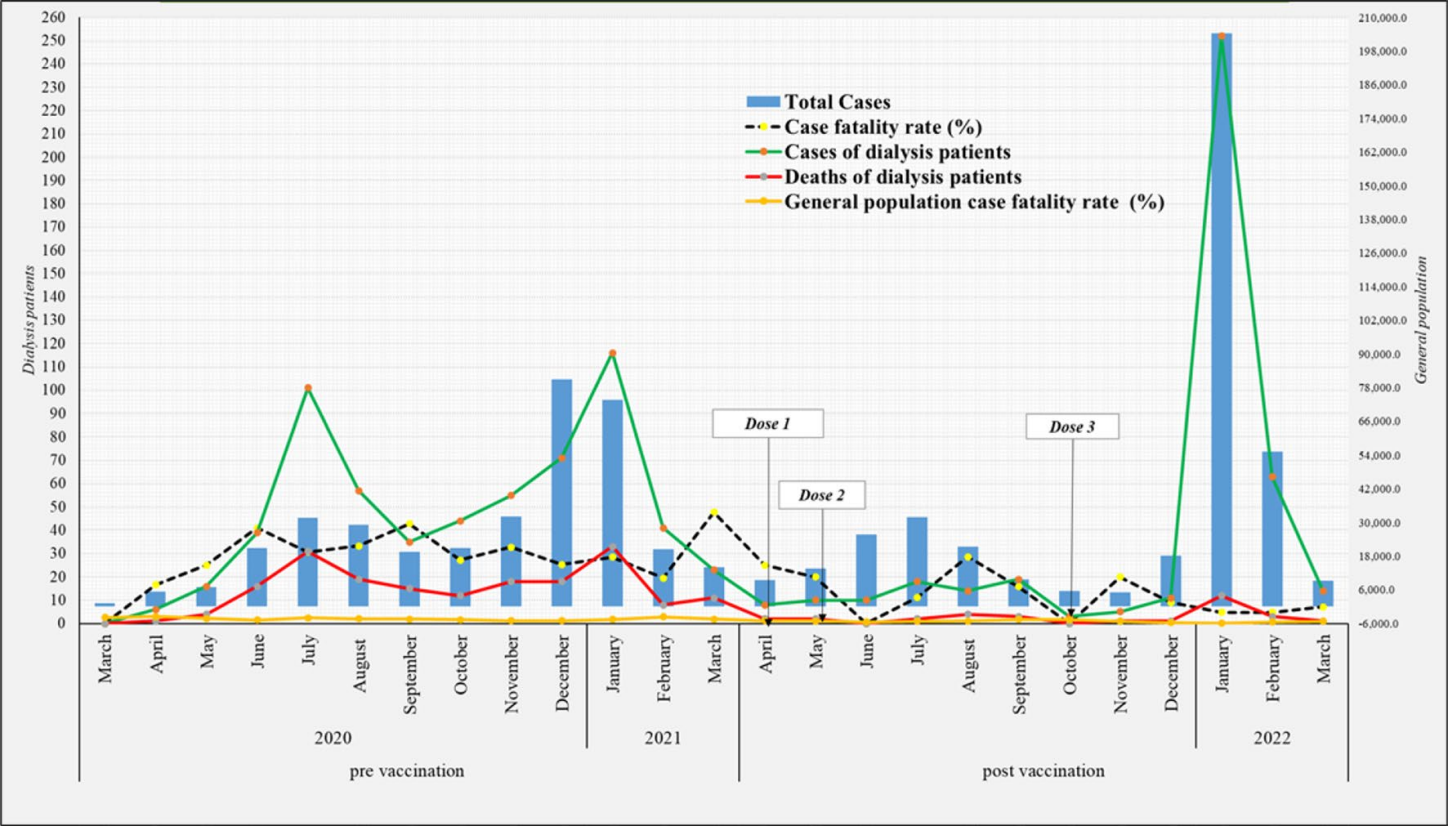
Total COVID-19 positive cases in dialysis patients vs. general population, March 2020–March 2022 Panama

Figure 2 shows total cases of COVID-19 positive patients in general population in Panama and in dialysis patients since March 2020 until March 2022, and with a differentiation for the period pre-vaccination and follow-up after three doses. The National Ministry of Health of Panama, following international guidelines recommendations, defined death by COVID-19 as a patient who presented at least one respiratory symptom, fever, and a specific positive PCR test, with no other cause of death at the time of the respiratory symptoms. Total deaths in dialysis patients for 2020 were 156 patients; 79 in 2021; and 25 for the first trimester of 2022. When comparing mortality among hemodialysis and peritoneal dialysis patients with COVID-19 infection from the study group, there is a significant difference (*p* 0.02) between hemodialysis patients (13/234) vs. peritoneal dialysis patients (6/27). Comparison of case fatalities for the period of 2020–2022 was 9.3% for dialysis patients against 0.2% for general population.

**Fig. 3:**
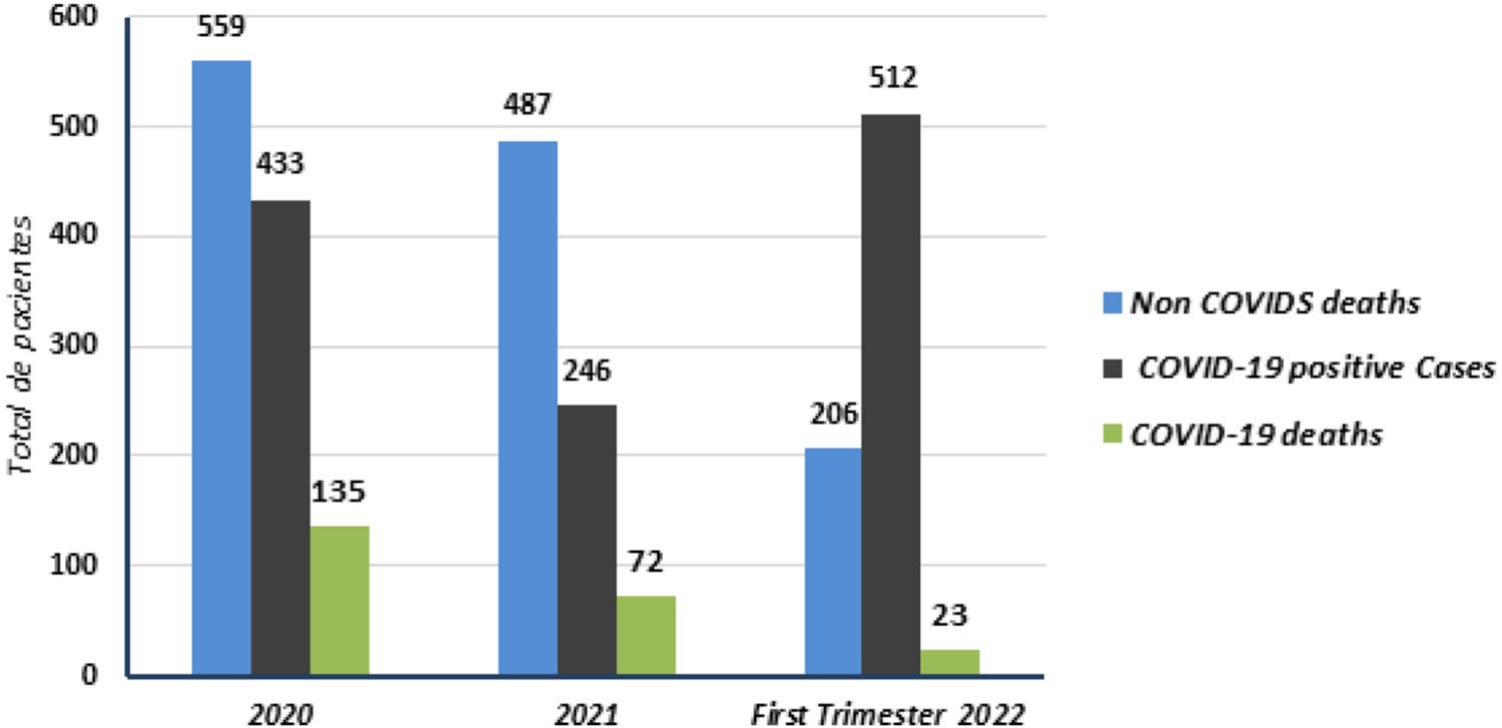
Total COVID-19 positive cases and deaths in dialysis patients in Social Security System 2020–2022

Total COVID-19 positive dialysis cases and deaths are shown in Fig. 3, and deaths due to other causes (cardiovascular and infectious non COVIDs), for the period of 2020, 2021, and first trimester of 2022 after vaccination period.

Table 2 shows the characteristics of dialysis patients diagnosed with COVID-19, before and after vaccination. In both groups, COVID-19 was significantly (*p* 0.0001) more frequent in men than in women, as in unvaccinated deceased patients; no significant difference was observed by sex in vaccinated deceased patients. Diabetes-hypertension or diabetes alone was the most frequent comorbidity in both groups. When comparing the period before availability of vaccines and after, in dialysis patients who died from COVID-19 infection in the country, fatality rate decreased from 30% in the unvaccinated group to 5% in the vaccinated group.

**Table 2:** Characteristics of dialysis COVID-19 infected patients before and after availability of vaccines

	Unvaccinated^a^	Vaccinated^b^
Positive COVID-19 patients	*n* = 459	*n* = 404
Age, median (years)	59	59.5
Sex	*p* 0.0001	*p* 0.0001
Male	316 (69%)	270 (67%)
Female	146 (31%)	134 (33%)
Comorbidities		
Diabetes- HTA	259 (56%)	208 (51%)
Diabetes	51 (11%)	95 (23%)
HTA	133 (29%)	80 (20%)
Other	13 (3%)	3 (1%)
NR	3 (1%)	18 (5%)
Deceased patients	136	19
Age, median (years)	62.5	64
Sex	*p* 0.0001	*p* 0.49
Male	91 (67%)	11 (58%)
Female	45 (33%)	8 (42%)
Comorbidities		
Diabetes HTA	87 (64%)	12 (63%)
Diabetes	16 (12%)	2 (10%)
HTA	28 (21%)	–
Other	3 (2%)	–
NR	–	5 (26%)

*HTA* Hypertension, *NR* not reported

^a^Unvaccinated: period from March 2020 to February 2021

^b^Vaccinated: period from March 2021 to March 2022

For evaluation of side effects, a subgroup of patients who reported both first and second questionnaire was analyzed and is described in Table 3. Local and systemic events were recorded, segregated by sex for 1409 patients. The study observed that women reported more local and systemic adverse effects (*p* < 0.0001) compared to men. When comparing between hemodialysis and peritoneal dialysis patients, there was no difference in symptoms in first dose, but with second dose, hemodialysis patients reported fewer symptoms than peritoneal dialysis patients (*p* < 0.0001).

**Table 3:** Classification of adverse effects after first and second dose, segregated by sex and dialysis modality for 1409 vaccinated patients

	Female *n* = 535	Male *n* = 874	*p* value
First dose			
Local	214 (40%)	274 (31%)	0.0001
Systemic	126 (24%)	155 (18%)	
Asymptomatic	195 (36%)	445 (51%)	
Second dose			
Local	195 (37%)	237 (27%)	0.0001
Systemic	132 (25%)	162 (19%)	
Asymptomatic	208 (38%)	475 (54%)	

	Hemodialysis *n* = 1162 *n* (%)	Peritoneal dialysis *n* = 247 *n* (%)	*p* value
First dose			
Local signs	414 (35.6)	99 (40.1)	0.19
No local signs	748 (64.4)	148 (59.9)	0.19
Systemic signs	253 (21.8)	48 (19.4)	0.44
No symptoms	909 (78.2)	199 (80.6)	0.44
Local + systemic	160 (13.8)	34 (13.8)	1
Second dose			
Local signs	361 (31)	98 (39.7)	0.01
No local signs	801 (69)	149 (60.3)	0.01
Systemic signs	242 (20.8)	77 (31.2)	0.0006
No symptoms	920 (79.2)	170 (68.8)	0.0006
Local + systemic	135 (11.6)	53 (21.5)	0.0001

Most common adverse effects are described in Table 4. No significant differences were observed in relation to adverse effects according to age group (*p* > 0.05). Fever was present in less than 11% of patients in first and 13% in second dose. Local (pain and redness) or systemic symptoms (fever, vomits, and diarrhea) were present in less than 12% of cases with both doses. For the third dose, evaluation of the 1140 questionnaires, 90% reported not having symptoms, 4% reported local adverse effects (pain and redness), and less than 5% reported systemic symptoms.

**Table 4:** Self-reported symptoms after each vaccine dose among dialysis participants

Type of symptoms	Symptoms after the first dose of the vaccine	Symptoms after the second dose of the vaccine	Symptoms after the third dose of the vaccine
Total patients	1725	1409	1140
No symptoms, *n* (%)	1000 (58)	1332 (94)	1034 (91)
Local pain (joint, head, in the vaccinated arm, muscle, throat), *n* (%)	466 (27)	30 (2)	44 (4)
Fever > 37.5° and other symptoms (headache, muscle pain, diarrhea), *n* (%)	190 (10.4)	14 (1)	28 (2)
Shortness of breath, *n* (%)	17 (1)	7 (0.4)	5 (0.5)
Others (vomiting, fatigue, insomnia, diarrhea, chills), *n* (%)	52 (3)	23 (1,6)	29 (2.5)

## Discussion

Panama had a population of 3,450,875 persons older than 18 years old as of March 2022 [9]. Distribution of population in the country is 51% female; dialysis population has a predominance of male patients (63%). Data of the National Authority for Government Innovation [7] reported that over 91% of people in the country received BNT162b2 Pfizer BionTech vaccine, both in dialysis and non-dialysis, since it was the first available vaccine in Panama [7]. The remaining 9% were vaccinated with ChAdOx1 nCoV-19 Aztra Zeneca´s vaccine.

In the COVID-19 vaccine trials done by the pharmaceutical companies, dialysis patients were not included, since these patients have a high prevalence of comorbidities and high cardiovascular mortality. There is much information developing from studies, and it has been described the added benefit of vaccination in dialysis patients, in reducing severity of illness from COVID-19 infection [10, 11].

Studies have shown that chronic kidney disease is a risk factor for high mortality following infection of SARS-CoV-2, due to older age and cardiovascular comorbidities [12]. Reports from studies from dialysis units in Europe found that older age, male gender, hypertension, diabetes, and glomerulonephritis were associated with an increased risk of mortality in COVID-19 infected patients [13]. In the dialysis patients, age was not associated with an increased risk when comparing unvaccinated with vaccinated patients. Fifty percent of our patients were more than 41 years of age, and 16% were in the oldest group of patients with more than 71 years, for both treatment modalities. There was no difference in sex in vaccinated deceased in comparison with unvaccinated patients. There was, however, a difference between frequency of cases, being more common in male patients than female.

During COVID-19 pandemic, peritoneal dialysis was considered an advantage over hemodialysis, because therapy in that group of patients was not interrupted and patients did not need to travel three times a week to receive in-center dialysis. PD patients represented an 18% of total dialysis patients in this study. When comparing mortality among vaccinated vs. unvaccinated dialysis patients, there was a difference (*p* 0.02) between hemodialysis vs. peritoneal dialysis patients. Some studies have demonstrated that PD patients had less COVID-19 infection rate than HD patients [14]. There are other reports indicating that mortality in PD patients was higher than HD patients, probably due to some comorbidities; in our case, PD patients had more diabetes, but not older age [15].

Vaccination coverage for first dose was similar between groups, with a 98% for dialysis patients and 95% for general population; 86% for second dose and 79.5% for the third dose in dialysis patients; and 77% for second dose and 39% for third dose for general population. Dialysis patients had a high vaccination rate, probably because awareness was made after publication of mortality data of patients with a 26% for the period pre-vaccination in the 2020 for patients in hemodialysis in Panama, higher than the 2% mortality for general population for that period [16]. Some studies have addressed that dialysis patients’ immune response to vaccination is worse than in general population [17], but some studies have demonstrated a robust immune response after a third dose [18]. This study was able to assess the importance of vaccination in dialysis patients, as lethality decreased from 30 to 5% once vaccination was available. Even though information about vaccine response is still in debate, because it is lower than in general population, probably because of a compromised immunity with delayed response in antibody production [19], for the moment, protections conferred by vaccines are important in decreasing severity and mortality. In Panamanian dialysis patients, mortality decreased after second and third dose, even though in the early 2022, genomic surveillance reports from Gorgas Institute reported the presence of Omicron variant in the country, that caused a rise in positive cases, with the higher peak of cases since the beginning of the pandemic, showing that the patients developed a strong response for Omicron variant [20].

An important consideration is that for the first time in the history of infectious pandemics, vaccines for COVID-19 infection were available in a short term, decreasing the rate of mortality in those countries where it was within reach. However, our patients still have a high rate of cardiovascular mortality or non-COVID-19-related infectious causes, such as those related to vascular access infections, which continues to be the number one cause of death among dialysis patients [21], that did not decrease during the pandemic.

When comparing local or systemic symptoms in dialysis patients, it was noted that women reported more symptoms than men. There are some biological differences, due in part by sex hormones, where female hormones make women exhibit a greater immune response and experience more adverse effects after vaccination than men [22]. Also, when comparing with studies with general population, systemic reactions reports were higher (59% first dose and 69% second dose) [23] than in this study with dialysis patients (24% female and 18% male first dose and 25% female and 19% male with second dose). Other studies have also observed a low reactogenicity in dialysis patients, explained by immune system senescence related to uremia immune deficiency [24]. Also, more symptoms were self-reported in the first questionnaire, than with other doses, and symptoms lessen with second and third dosage, probably due to better tolerance of pain, for example, or secondary to knowledge of the previous experience. None of the reported adverse events were severe.

This study has limitations. Despite being a national study and one that managed to recruit a good number of patients, it could not use all of them, probably due to the issue of fear of COVID and isolation measures before patients had the availability of vaccines. Our data on reference population were limited only to sex, types of vaccines, vaccination doses, and mortality. The system does not have detailed information of general population available. This study is important for the countries in the region, where the ethnic conformation does not have a majority of Caucasians as in the large number of published studies.

## Conclusion

Infection with COVID-19 had a higher prevalence and mortality among hemodialysis and dialysis patients compared to the general population in Panama. As new variants emerge, studies will need to determine how many boosters and how frequent dialysis patients will need to be vaccinated, but for the moment, this study’s data support the use of vaccines in dialysis patients, since they are associated with a decrease rate of hospitalization for severe infection and deaths, and are safe, with a low rate of adverse effects and no severe adverse events reported.

## Data Availability

All data generated or analysed during this study are included in this published article.
